# Design, Development and Testing of a Low-Cost sEMG System and Its Use in Recording Muscle Activity in Human Gait

**DOI:** 10.3390/s140508235

**Published:** 2014-05-07

**Authors:** Tamara Grujic Supuk, Ana Kuzmanic Skelin, Maja Cic

**Affiliations:** Faculty of Electrical Engineering, Mechanical Engineering and Naval Architecture, Laboratory of Biomechanics and Automatic Control Systems, R. Boskovica 32, Split 21000, Croatia; E-Mails: akuzmani@fesb.hr (A.K.S.); maja@fesb.hr (M.C.)

**Keywords:** surface electromyography (sEMG) signals, biomedical sensors, bioamplifier, artificial EMG signal, noise removal, wavelets, EMG signal envelope, muscle activity during human gait

## Abstract

Surface electromyography (sEMG) is an important measurement technique used in biomechanical, rehabilitation and sport environments. In this article the design, development and testing of a low-cost wearable sEMG system are described. The hardware architecture consists of a two-cascade small-sized bioamplifier with a total gain of 2,000 and band-pass of 3 to 500 Hz. The sampling frequency of the system is 1,000 Hz. Since real measured EMG signals are usually corrupted by various types of noises (motion artifacts, white noise and electromagnetic noise present at 50 Hz and higher harmonics), we have tested several denoising techniques, both on artificial and measured EMG signals. Results showed that a wavelet—based technique implementing Daubechies5 wavelet and soft sqtwolog thresholding is the most appropriate for EMG signals denoising. To test the system performance, EMG activities of six dominant muscles of ten healthy subjects during gait were measured (gluteus maximus, biceps femoris, sartorius, rectus femoris, tibialis anterior and medial gastrocnemius). The obtained EMG envelopes presented against the duration of gait cycle were compared favourably with the EMG data available in the literature, suggesting that the proposed system is suitable for a wide range of applications in biomechanics.

## 1. Introduction

The precise measurement and analysis of human movements and muscle activity are an essential step in biomechanical research in medicine, rehabilitation, and sport. Measurement systems used for motion tracking include highly sophisticated 3D motion-capturing systems, as well as electromyography (EMG) systems which record muscle activity during motion. Nowadays many commercial systems are available for precise measurements of human motion and muscle activity represented by EMG signals. In the context of tracking the movements of a human body, optoelectronic devices are the number-one tool. Optoelectronic devices typically use small markers that are attached to a subject's body surface, and a set of two or more cameras is used for capturing the markers' motions [1–3]. Highly sophisticated commercial systems such as Vicon [4] (which uses reflective passive markers) or Optotrak [5] (which uses active markers) are often considered as a gold standard in human motion analysis. In the context of tracking muscle activity during movements, commercially available, highly precise, and high quality EMG systems are manufactured by Motion Lab Systems, Inc. [6], BTS Bioengineering [7], Delsys [8], *etc*.

### 1.1. Related Work Dealing with Electromyography (EMG) Signal Processing

Electromyography (EMG) refers to a collective electric signal from muscles, which is controlled by the nervous system and produced during muscle contraction [9]. The signal represents the anatomical and physiological properties of muscles; in fact, a surface EMG signal is the electrical activity of an underlying muscle [10]. EMG signals are becoming increasingly important in many applications, including biomechanical, clinical/biomedical, prosthesis or rehabilitation devices, human machine interactions, and more [9]. For example, EMG signals can be used to generate device control commands for rehabilitation equipment such as robotic prostheses, and have been deployed in many clinical and industrial applications [11].

The detection of electromyographic signals is a very complex process, which is affected not only by muscle anatomy and the physiological process responsible for signal generation but also by external factors and different types of noises, such as the inherent noise of the hardware employed in signal amplification and digitalisation [12]. Therefore, it is very difficult to remove the noises from recorded EMG signals efficiently. Most common noises in EMG signals are inherent in the electronic equipment and motion artifacts, and can be electromagnetic noise or cross-talk.

Inherent noise or white noise is generated by electronic equipment employed for EMG signal recording and the frequency components of this noise range from direct current (DC) to several thousand Hz [9]. Motion artifacts are noises with a frequency range from 1 Hz to around 15 Hz, and have a voltage comparable to the amplitude of an EMG signal. These noises are introduced by the interface between the detection surface of the electrode and the skin, and the movement of the cable connecting the electrode to the amplifier.

Electromagnetic noise, present at 50 (60) Hz frequency and higher harmonics, corrupts EMG signals since the human body behaves like an antenna: the surface of the body is continuously inundated with electric radiation [9]. Because the power line radiation (50 or 60 Hz) is a dominant source of electrical noise, it is tempting to design devices that have a notch-filter at this frequency. Theoretically, this type of filter would only remove the unwanted power line frequency, however, practical implementations also remove portions of the adjacent frequency components. Therefore, according to De Luca [13], because the dominant energy of the EMG signal is located in the 20–400 Hz range, the use of notch filters is not advisable when there are alternative methods of dealing with the power line radiation such as digital signal processing and denoising techniques.

Cross-talk represents an undesired EMG signal from a muscle group which surrounds the muscle of interest. Therefore, the electrical activity of surrounding muscles interferes with the activity of the recorded muscle. Since cross-talk is easily recorded from undesired muscles, it is actually very difficult to avoid this type of noise.

Researchers have made strenuous efforts to solve the problem of EMG signal denoising [14–18]. Various digital signal processing techniques are employed, from classical digital filters to modern filtering techniques such as wavelets. Conforto and colleagues [19] tested several filtering procedures to reject the motion artifact from EMG signals. They tested the moving average filter, the moving median filter, eighth-order Chebyshev high pass filters with a cut-off frequency of 20 Hz, and the adaptive filter based on orthogonal Meyer wavelets. They found that wavelet-based filtering provides the best results. Also, many researchers have reported good results for EMG signal processing and analysis [9] by employing different advanced algorithms, such as Wigner-Ville distribution, independent component analysis, empirical mode decomposition [20], and the Hilbert spectrum [21].

### 1.2. Requirements for Electromyography (EMG) System Design

An important issue regarding the design of an EMG system is determining an appropriate design for the bioamplifier. The first condition imposed is that the bioamplifiers should be small in size and weight, in order to be wearable on humans while performing the movements. Secondly, bioelectric amplifiers require a high gain level, a low density of equivalent input noise, a high common mode rejection ratio (CMRR) and a high impedance input [22,23]. Most of these features can be achieved by using a monolithic instrumentation amplifier (IA) as a front stage [22]. Since the required gain for EMG amplifiers is at least 1,000, this gain cannot be achieved in a single stage because of output saturation issues. Therefore, the gain of the front instrumentation amplifier should be around 100, and the additional gain should be accomplished by the second stage of amplification, usually by means of the operational amplifier. EMG bioamplifiers should also be designed as filters, to reject direct current offset and to serve as anti-aliasing filters.

Modern commercial multi-channel EMG systems available on the market offer a wide variety of possibilities for high-quality recording of EMG signals. They offer highly accurate amplifiers with adjustable gains, high sampling frequencies, wireless data transmission, and active electrodes by means that the preamplifier is inserted into the same housing with recording surfaces. Home-made EMG systems could hardly compete with the available commercial versions. But, the main drawback of all commercial EMG systems is their very high cost, varying from several tens of thousands of euros, thus making these systems beyond the reach of many human-motion research laboratories.

Therefore, one of the research focuses of our Laboratory of Biomechanics, Automatic and Control Systems at the University of Split is the design, development, and evaluation of low-cost human-motion measurement systems. We designed an optical motion-tracking system based on active white light markers [24] and showed that it could be used as an efficient tool for measuring kinematic data on human motion. We also developed a structured light 3D scanner for estimation of human anthropometric parameters [25]. The next step in our research was the design, development and evaluation of a low-cost sEMG system, which is presented in this paper. We described in detail how to design a low-cost EMG system, applicable for various measurements of muscle activity during human motions. Special emphasis is placed on the design of a small-sized two-stage bioamplifier and on a signal-denoising technique based on wavelets. We tested our system by measuring muscle activity of six muscles during human gait, and the results showed that the proposed system can serve as an muscle-activity recording tool in various biomechanical applications.

## 2. sEMG System Prototype Design Overview

**Figure 1.**
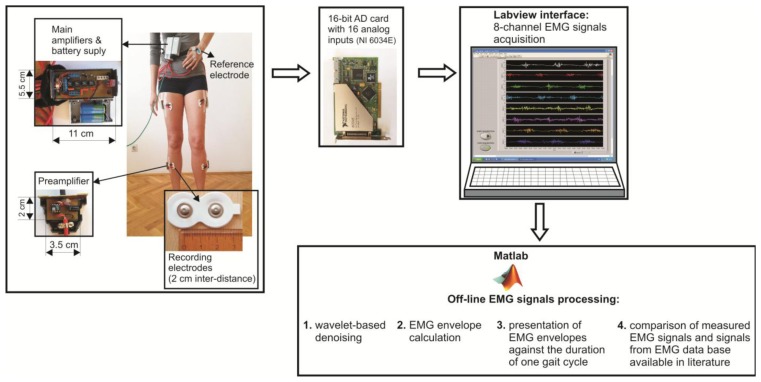
Flowchart of the prototype of surface electromyography (sEMG) system and EMG signal processing techniques.

The flowchart of the prototype of eight channel sEMG system and EMG signal processing techniques is illustrated in Figure 1.

The surface electrodes used for EMG recording were commercially available concentric Ag-AgCl electrodes, type Noraxon Dual Electrodes, and inter-electrode distance was 2 cm. Since the electromyograms are low amplitude signals (from several μV up to 4–5 mV), with 90%–95% of total EMG power present within frequency range from 20 Hz to 400 Hz, and very sensitive to noises, the focus should be on the design of a low-noise, accurate bioamplifier. We used a two-cascade circuit (preamplifier and the main amplifier). Physically, eight preamplifiers were placed into eight separated, plastic and shielded housings, and connected to the electrodes with short, shielded cable, 3.5 cm in length. Eight main amplifiers, together with two power supply batteries (±9 V), were integrated into the shared shielded housing placed on the subject's belt (Figure 1). In order to determine the duration of gait cycle while the subject was walking, we taped the switch to the subject's bare foot (heel). Therefore, each heel-ground contact was recorded by the increasing slope of the rectangular impulse signal.

The amplified EMG signals were passed to the 16-bit A/D converter and personal computer via a 10 m long shielded cable. The A/D sampling frequency of EMG signals was 1000 Hz. A Labview-based user interface was designed to collect the data and display raw sEMG signals on a monitor. Offline sEMG signal processing and analysis were performed with software written in Matlab (The MathWorks, Natick, MA, USA).

### 2.1. Bioamplifier Design

**Figure 2.**
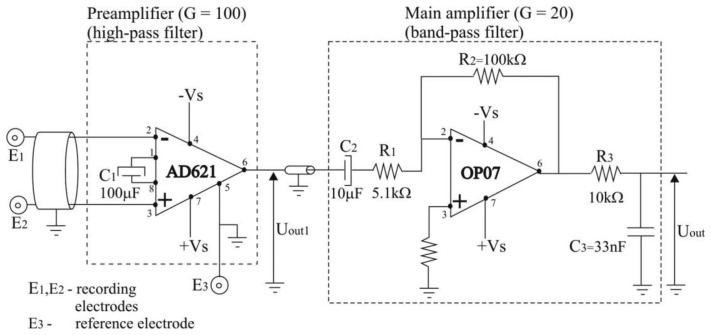
The electric scheme of the bioamplifier consisted of two stages: the preamplifier and the main amplifier.

The electric scheme of the bioamplifier is presented in Figure 2. It was designed as a two-cascade circuit (preamplifier and main amplifier), with total amplification of *G* = 2,000, and a bandwidth of 3 Hz to 500 Hz.

#### 2.1.1. Preamplifier Design

In order to obtain wearable preamplifier, which should be as small in size and as low in weight as possible, so it can be taped to the human skin by adhesive tape, we used only two electronic components for preamplifier design: instrumentation amplifier AD621 and capacitor, *C*_1_ = 100 μF, as depicted in Figure 2. Instrumentation amplifier AD621, manufactured by Analog Devices (Norwood, MA, USA) [26] was chosen because it is an easy-to-use, low-cost, low-power, highly accurate instrumentation amplifier ideally suited for precise measurement of low amplitude signals such as EMG, and for design of battery-powered and portable equipment such as proposed EMG system. AD621 has very high input impedance of 10 GΩ in parallel with 2 pF, as required for recording of EMG signals, and wide power supply range from ±2.3 V to ±18 V. When operating from high source impedances, as in EMG or electrocardiographic (ECG) recording, and blood pressure monitors, the AD621 features the good combination of low noise and low input bias currents. Voltage noise is specified as 9 nV/√*Hz* at 1 kHz and 0.28 μV p-p from 0.1 Hz to 10 Hz. Input current noise is also extremely low at 0.1 pA/√*Hz*. The common mode rejection ratio (CMRR) of AD621 has a high value of 130 dB for frequency interval of 0.1 Hz to 10 Hz, then drops and reaches the value of 90 dB at 1,000 Hz. The CMRR at frequency of 50 Hz has a value of app. 120 dB. The high values of CMRR ensure an optimal rejection of common mode input signals. The power supply rejection ratio (PSRR) of AD621 has a high value of 140 dB for frequency interval of 0.1 Hz to 10 Hz, then drops and reaches the value of 110 dB at 1,000 Hz. For more information about AD621 theory of operation and circuit topology we refer the interested reader to AD621 data sheet [26]. Capacitor used was aluminium solid electrolytic capacitor, manufactured by Jamicon, rated voltage of 25 V, and capacitance tolerance of ±20%.

**Figure 3.**
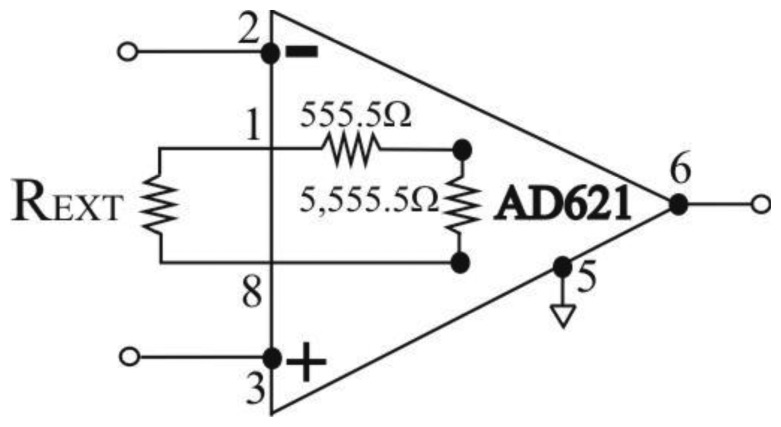
AD621 instrumentation amplifier: Gain setting.

The AD621 amplifier gain, *G*, is given by Equation (1), available in the AD621 data sheet [26]:

(1) $$G=\frac{50\text{k}\Omega }{R_{G}}+1$$

where R_G_ is the parallel combination of resistors (see Figure 3):

(2) $$R_{G}=5555.5\|(555.5+R_{\text{EXT}})(\Omega )$$

Therefore, amplifier gain can be programmed for any gain between 10 and 100 by connecting a single external resistor, *R*_EXT_, between pins 1 and 8 (Figure 3). By combining Equations (1) and (2) we can express the dependence between gain *G* and *R*_EXT_ as:

(3) $$G=\frac{9(R_{\text{EXT}}+6111)}{\left(R_{\text{EXT}}+555.5\right)}+1$$

In order to achieve the gain value *G* = 100, but only for higher frequencies, we connected a capacitor (*C*_1_ = 100 μF) between pins 1 and 8 (see Figure 2). In that case, gain *G* expressed by Equation (1) will become:

(4) $$G=\frac{50\text{k}\Omega }{Z_{G}}+1$$

where *Z_G_* is frequency-dependent impedance which replaces resistance *R_G_* from Equation (2), and which can be expressed as:

(5) $$Z_{G}=5555.5\|(555.5+\frac{1}{C_{1}j\omega })=5555.5\|(555.5+\frac{10^{4}}{j\omega })(\Omega )$$

By combining Equations (4) and (5), we can express the gain *G* by the following equation (Figure 2):

(6) $$G=\frac{U_{\text{OUT}1}}{U_{\text{IN}}}=\frac{U_{\text{OUT}1}}{U_{E1}-U_{E2}}=\frac{9(\frac{10^{4}}{\omega j}+6111)}{\frac{10^{4}}{\omega j}+555.5}+1=\frac{10(1+0.555\omega j)}{1+0.0555\omega j}$$

In this case, the gain of preamplifier is: *G* = 100 = 40 dB, and the cut-off frequency is:

(7) $$f_{\text{cut}\_\text{off}}=\frac{1}{2\pi }*\omega_{\text{cut}\_\text{off}}=\frac{1}{2\pi }*\frac{1}{0.0555}\text{Hz}=2.8\text{Hz}≅3\text{Hz}$$

**Figure 4.**
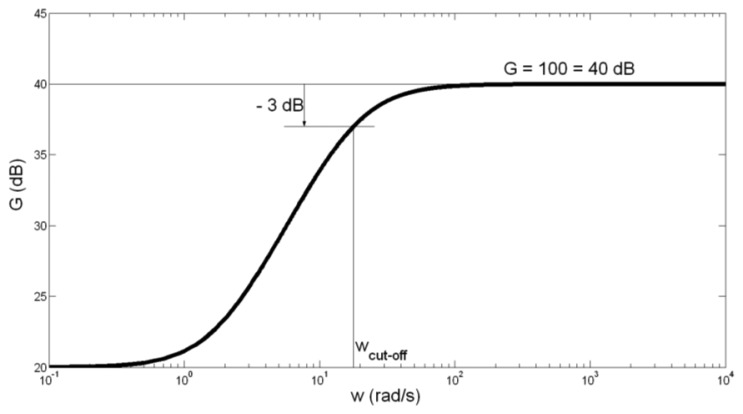
Amplitude-frequency characteristic of the preamplifier.

The amplitude-frequency characteristic of the preamplifier is presented in Figure 4.

Having in mind somewhat high tolerance of capacitor *C*_1_ capacitance of ±20%, we have calculated the border values of *f*_cut-off_. For *C*_1_ = 100 μF + 20% = 120 μF, *f*_cut-off_ is obtained to be 2.38 Hz. For *C*_1_ = 100 μF − 20% = 80 μF, *f*_cut_off_ is obtained to be 3.58 Hz. We can conclude that the possibly spread of *f*_cut-off_ between values 2.38 Hz and 3.58 Hz does not influence significantly on the preamplifier function.

#### 2.1.2. Main Amplifier Design

For the second cascade of the bioamplifier (main amplifier) we used an operational amplifier, OP07, manufactured by Analog Devices Inc. (Norwood, MA, USA) [27], resistors and capacitors (Figure 2). The CMRR of OP07 has a high value of 120 dB. The PSRR is 5 μV/V. Capacitor *C*_2_ = 10 μF was aluminium solid electrolytic capacitor, manufactured by Jamicon (New Taipei City, Taiwan) with a rated voltage of 250 V, and capacitance tolerance of ±20%. Capacitor *C*_3_ = 33 pF was metallized polyester film non-polarized capacitor, manufactured by BC Components (Malvern, PA, USA), rated voltage of 63 V, and capacitance tolerance of ±5%. The general purpose of the main amplifier is to further amplify EMG signals to a level compatible with the analog/digital (AD) converter, eliminate direct current (DC) offset, and act as an anti-aliasing filter. Therefore, we constructed the main amplifier as a band-pass filter, with the gain *G* = 20, and cut-off frequencies of *f*_cut_off_low_ = 3.13 Hz ≈ 3 Hz, and *f*_cut_off_high_ = 482 Hz ≈ 500 Hz. High cut-off frequency was set to 500 Hz, in consideration of Nyquist sampling theory and EMG signal sampling frequency of 1,000 Hz. The frequency- dependent gain, *G* of the main amplifier, can be expressed as (Figure 2):

(8) $$G=\frac{U_{\text{OUT}}}{U_{\text{OUT}1}}=-\frac{R_{2}}{R_{1}+\frac{1}{C_{2}\omega j}}\cdot \frac{\frac{1}{C_{3}\omega j}}{R_{3}+\frac{1}{C_{3}\omega j}}=-\frac{\omega j}{1+\frac{5.1}{100}\omega j}\cdot \frac{1}{1+\frac{33}{10^{5}}\omega j}$$

**Figure 5.**
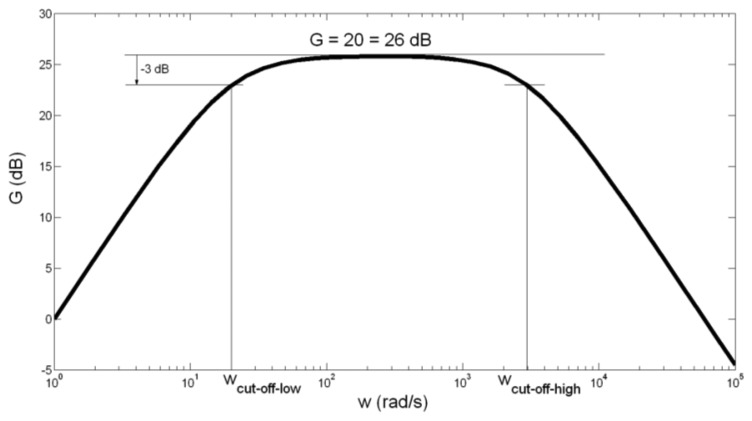
Amplitude-frequency characteristic of the main amplifier acting as a band-pass filter.

The amplitude-frequency characteristic of the main amplifier is presented in Figure 5.

As seen in Figure 5, band-pass gain is *G* = 20 = 26 dB, and cut-off frequencies are:

(9) $$\begin{array}{ll} f_{\text{cut}\_\text{off}\_\text{low}}=\frac{1}{2\pi }\cdot \omega_{\text{cut}\_\text{off}\_\text{low}} & =\frac{1}{2\pi }\cdot \frac{1}{R_{1}C_{2}}\text{Hz}=\frac{1}{2\pi }\cdot \frac{100}{5.1}=2.8\text{H}z≅3\text{Hz}\\ f_{\text{cut}\_\text{off}\_\text{high}}=\frac{1}{2\pi }\cdot \omega_{\text{cut}\_\text{off}\_\text{high}} & =\frac{1}{2\pi }\cdot \frac{1}{R_{3}C_{3}}\text{Hz}=\frac{1}{2\pi }\cdot \frac{10^{5}}{33}=482\text{Hz}≅500\text{Hz} \end{array}$$

The band-pass filter has somewhat gentle roll-off of 20 dB/dec. We made this choice by following the guidelines for detection and recording EMG signals provided by De Luca in his report [13] where he suggested the judicious filtering with a roll-off of 12 dB/oct.

Regarding the somewhat high tolerance of capacitor *C*_2_ capacitance of ±20%, we have calculated the border values of *f*_cut-off-low_. For *C*_2_ = 10 μF + 20% = 12 μF, *f*_cut-off-low_ is obtained to be 2.6 Hz. For *C*_2_ = 10 μF − 20% = 8 μF, *f*_cut-off-low_ is obtained to be 3.9 Hz. As in case of preamplifier, we can conclude that the possibly spread of *f*_cut-off-low_ between values 2.6 Hz and 3.9 Hz does not significantly influence the function of the main amplifier.

### 2.2. EMG Signal A/D Conversion and Labview—Based Acquisition

For A/D conversion of recorded and amplified EMG signal we used 16 bit A/D card with 16 analog inputs, type NI 6034E, manufactured by National Instruments (Dallas, TX, USA) [28]. The full scale of AD can be selected; we used the range of ±5 V, since our measured and rectified EMG signals did not exceed the amplitude of ±5 V. The resolution of the AD card with the selected full scale range is:

(10) $$\text{Resolution}=\frac{10\text{V}(\text{peak to peak})}{2^{16}}=0.15259\text{mV}$$

Raw EMG signals' recording was done in Labview. We made simple Labview interface which can record EMG signals from eight channels and save them in *.lvm files. Those files have ASCII format and can be easily imported into Matlab. Therefore, we imported recorded EMG signals from Labview to Matlab, and performed off-line processing, as depicted in Figure 1.

## 3. EMG Signal Processing

In this section the detailed workflow of digital processing performed on EMG signals is described. Since we were focused on wavelet-based denoising, a short overview of discrete wavelet transformation (DWT) is presented. Afterwards, the algorithms for DWT-based white noise and motion artifact removal are described. In order to reach the decision which wavelet and which threshold would be the most appropriate to apply for EMG signals denoising, we simulated an artificial EMG signal with added white noise and motion artifact, and with known signal to noise ratio (SNR). Several different wavelets and classical digital filtering procedures were tested. From the obtained results, we could decide which wavelet is the most appropriate for EMG signals denoising. After we have denoised measured EMG signals by using the chosen wavelet, we rectified signals, and then we calculated EMG signal envelopes. Envelopes were averaged over all measured subjects, and the trajectory of average envelope for each measured muscle was presented against the duration of one gait cycle. Finally, we compared our signals envelopes with the data available in literature [29].

### 3.1. Discrete Wavelet Transformation, DWT

The algorithm of discrete wavelet transformation, DWT, uses digital filtering techniques in order to obtain a time-scale representation of a digital signal. DWT employs two sets of functions, called scaling functions ϕ(*t*) and wavelet functions ψ(*t*), which are associated with low-pass filters *h*(*n*) and high-pass filters *g*(*n*) through the following expressions [30,31]:

(11) $$\phi (t)=2\sum_{n=0}^{N-1}h(n)\phi (2t-n)$$

(12) $$\psi (t)=2\sum_{n=0}^{N-1}g(n)\phi (2t-n)$$

**Figure 6.**
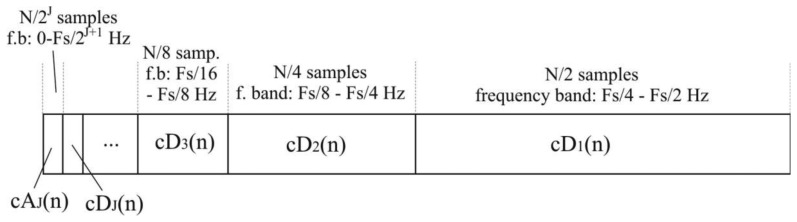
Wavelet coefficients concatenated into matrix.

The decomposition of the signal into different frequency bands is obtained by successive high-pass and low-pass filtering of time domain signals, followed by downsampling by two. The two filtering and downsampling operations can be expressed by:

(13) $$cA_{i}(k)=\sum_{n}cA_{i-1}(n)h(2k-n)$$

(14) $$cD_{i}(k)=\sum_{n}cA_{i-1}(n)g(2k-n)$$

where *cA_i_* describes approximation coefficients and *cD_i_* describes detail coefficients of *i*-th level. In summary, the result of *J*-th level DWT performed on *N* samples of digital signals sampled with frequency *F*_s_ is a set of detail coefficients *cD_i_*, *i* = 1 to *J* and approximation coefficients *cA_J_*, concatenated into a single matrix of length *N*, as shown in Figure 6.

The total number of *N*/2*^i^* detail coefficients of *i*-th level, *cD_i_*, represents the original signal in a frequency band of [*F*_s_/2*^i^*^+1^-*F*_s_/2*^i^*] Hz. *N*/2*^J^* samples of approximation coefficients, *cA_J_*, represent signals in the lowest frequency band between 0 and *F*_s_/2*^J^*^+1^ Hz.

The procedure of signal decomposition is followed in reverse order for signal reconstruction or inverse DWT. The coefficients at every level are upsampled by two, passed through the synthesis filters *h*′(*n*) and *g*′(*n*) (low-pass and high-pass, respectively) and then added. The analysis and synthesis filters are known as quadrature mirror filters, QMF. The reconstruction formula for each level becomes:

(15) $$cA_{i}(k)=\sum_{n}\left(cA_{i+1}(n)h'(2k-n\right)+cD_{i+1}(n)g'(2k-n))$$

and the reconstructed signal is:

(16) $$x(k)=\sum_{n}\left(cA_{1}(n)h'(2k-n\right)+cD_{1}(n)g'(2k-n))$$

### 3.2. White Noise Removal by DWT and Thresholding

The thresholding approach to wavelet-based noise removal, WBNR, was developed by Donoho and colleagues [32–34]. This method, which removes Gaussian white noise from a signal, relies on the following principle: wavelet transforms compress the energy of a noise-free signal into a small number of large coefficients called “true” signal coefficients. As a consequence of the higher energy of a noisy signal, more wavelet coefficients are of a relatively larger (non-zero) magnitude, but much smaller than the “true” signal coefficients. These coefficients, contributed by the noise, can then be identified and thresholded, and the reconstruction yields a cleaned, de-noised version of the signal. The general wavelet denoising procedure we used is as follows [35]:

- Choose the appropriate wavelet for signal denoising
- Apply DWT to the noisy signal to produce the noisy wavelet coefficients.
- Select an appropriate threshold limit at each level and the threshold method (hard or soft thresholding) which best removes the noise.
- Perform inverse wavelet transformation of the thresholded coefficients to obtain a denoised signal.

### 3.3. Motion Artifact Removal by Wavelets

EMG signals usually contain low-frequency noise ranging from 0 to ∼15 Hz, as mentioned earlier. In this work the following procedure for artifact removal was implemented:

- Prior to the decomposition of EMG signals by DWT, the level of decomposition, *J*, is calculated as follows:
  (17) $$\frac{F_{\text{s}}}{2^{J+1}}\approx f_{\text{noise}}$$
  As the sampling frequency *F*_s_ is 1,000 Hz and *f*_noise_ is 15 Hz, *J* is obtained to be 5.
- DWT of *J*-th level is performed. Approximation coefficients are on the fifth level, and *cA*_5_ represents EMG signals in the frequency range of 0 to *F*_s_/2^6^ ≈ 15 Hz. Therefore, these coefficients represent artifacts and are removed (replaced by zero values) in order to clean the signal from the artifact. Detail coefficients are left unchanged.
- Performing of signal reconstruction.

### 3.4. Simulation of Artificial EMG Signal for Testing of Denoising Algorithms

**Figure 7.**
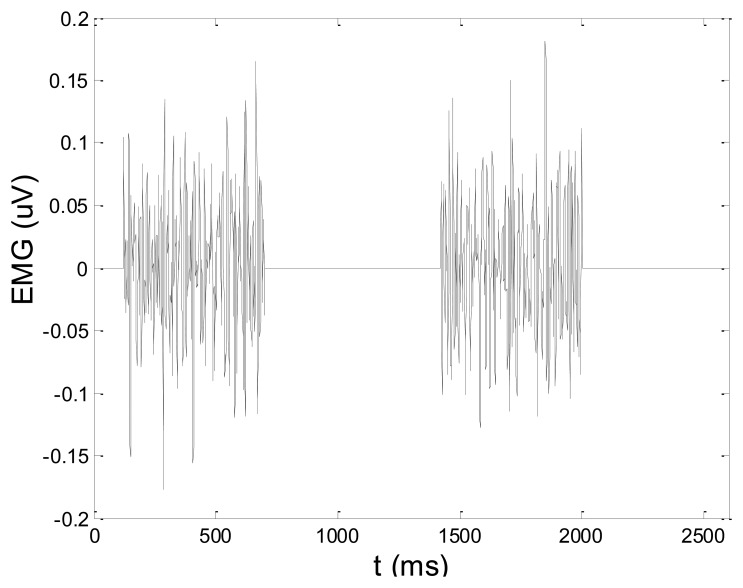
Simulated artificial EMG signal.

**Figure 8.**
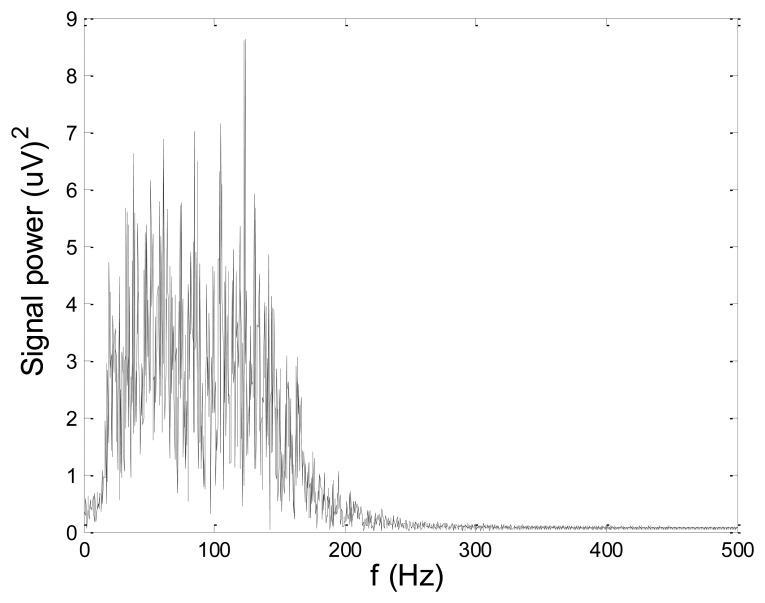
Power spectrum of simulated artificial EMG signal.

**Figure 9.**
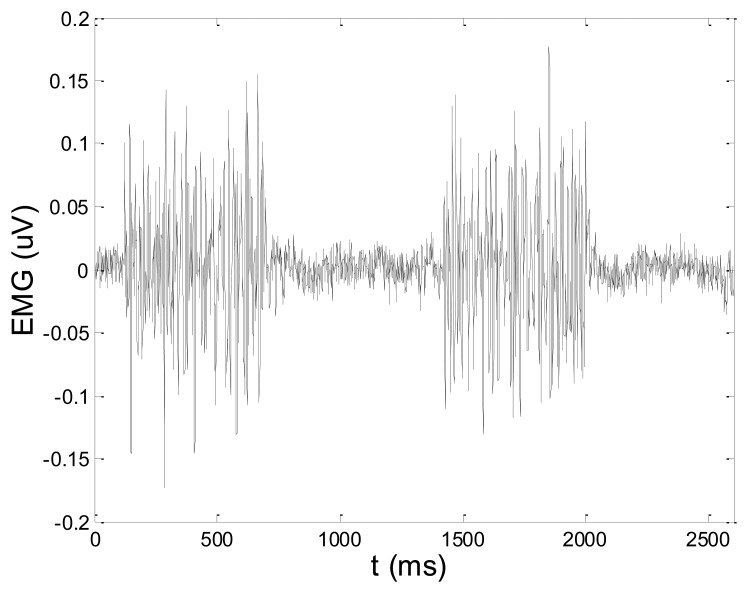
Simulated artificial EMG signal with added noises.

**Figure 10.**
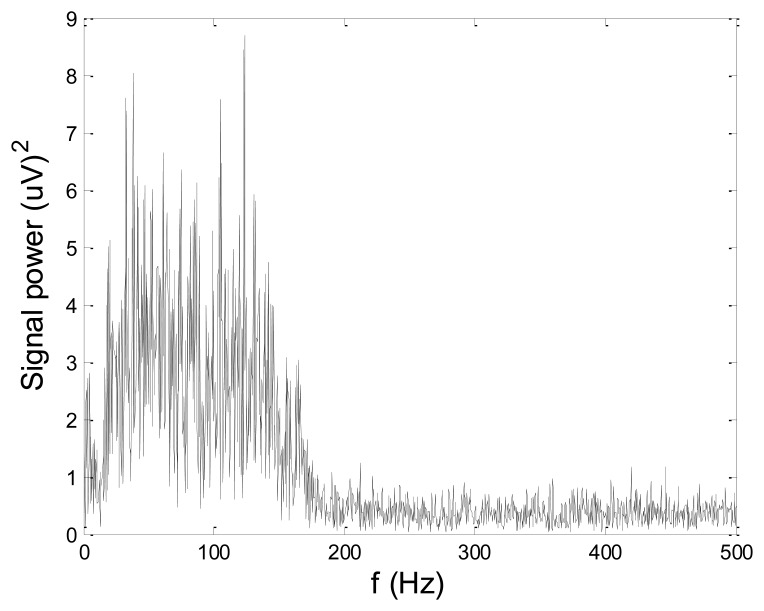
Power spectrum of simulated artificial EMG signal with added noises.

In order to test wavelet based noise removal techniques and compare them to classical filtering methods, we have simulated artificial EMG signal, shown in Figure 7. Gaussian white noise, *N* ∼ (0,1), was filtered by 4th order Butterworth filter with 20–150 Hz bandwidth, in order to obtain frequency spectrum similar to EMG signal. Amplitude of the signal was modulated to obtain two active intervals, separated by resting intervals with no EMG activity. Sampling frequency was 1,000 Hz and the time duration of the signal was 2.6 s. The frequency spectrum of the artificial signal is presented in Figure 8. In order to obtain a noised signal, simulated EMG signal was contaminated by normally distributed white noise and an artifact pattern extracted from a real EMG signal measured on the tibialis anterior muscle during gait. The so-obtained noised EMG signal, shown in Figure 9, consisted of two different zones: a burst zone where noises and myoelectric activity coexist, and an inter-burst zone where only the noise contribution is present. The frequency spectrum of a noised simulated signal is shown in Figure 10.

The efficacy of denoising techniques was determined by calculation of signal to noise ratio (SNR) and root mean square error (RMSE), according to the following formulas:

(18) $$\text{SNR}(\text{dB})=10\log_{10}\left(\frac{\sum_{n=1}^{N}x{\left[n\right]}^{2}}{\sum_{n=1}^{N}{\left(\hat{x}\left[n\right]-x\left[n\right]\right)}^{2}}\right)$$

(19) $$\text{RMSE}=\sqrt{\frac{1}{N}\sum_{n=1}^{N}{\left(\hat{x}[n]-x[n]\right)}^{2}}$$

where:

*x*[*n*]: simulated artificial EMG signal, shown in Figure 7
*x̂*[*n*]: noisy signal (shown in Figure 9), or denoised signal, depending on the context
*N*: number of signal samples (in all test cases, *N* = 2,600)

The SNR for simulated artificial EMG signal with added noises is obtained to be 7.9 dB. For testing the wavelet-based denoising techniques, DWT on 5-th level was performed, and some of the used wavelets were: Daubechies5 (db5), Daubechies8 (db8), Symmlet8 (sym8), Coifflet5 (coif5), and Discrete Meyer (dmey). Motion artifact is filtered by elimination of approximation coefficients of the 5th level. White noise is filtered by thresholding detail coefficients of levels 1 to 5, by employing soft and hard version of sqtwolog, minimaxi, rigrsure and heursure thresholds, implemented in Matlab's Wavelet Toolbox [35]. Standard band-pass filters used for comparison with wavelet—based techniques were an eight order Butterworth filter and an eight order Chebyshev. Filter's coefficients were calculated for band pass ranging from 15 to 200 Hz. Denoising results are presented in Section 5.1.

### 3.5. EMG Signal Envelope Calculation

In biomechanical applications, EMG signals are usually expressed by envelopes. In this paper, measured and wavelet-based denoised EMG signals were rectified and filtered by a moving average filter with a moving window consisting of *N* = 100 samples, in order to obtain linear envelopes of signals.

### 3.6. Comparison of Measured EMG Signals with Existing EMG Data Base

Winter provided in his book [29] a database of EMG signals recorded on twenty five leg muscles. For recording, he used concentric electrodes (inter-electrode distance was 2 cm), and thoroughly described the electrodes positions on each muscle. After recording, he rectified EMG signals, calculated envelopes of each signal, and then calculated averaged envelope trajectories over all measured subjects. He presented in Figures EMG averaged envelopes against the percentage of one gait cycle (from 0% to 100% of gait cycle). There are two main phases in the gait cycle, stance and swing phase [36]. The stance phase begins at 0% of gait cycle, with heel-ground contact and ends when the foot leaves the ground (toe-off) at 60% of gait cycle. Therefore, during the stance phase (0%–60% of gait cycle), the foot is on the ground. During the swing phase (60%–100% of gait cycle) the same foot is no longer in contact with the ground and the leg is swinging through in preparation for the next foot strike, when the subsequent gait cycle begins.

Besides EMG envelope graphical presentations against one gait cycle, Winter also included tables with numerical values of EMG envelopes amplitudes for each 2% of gait cycle, thus providing in total 51 samples of EMG signal amplitudes for each muscle. Therefore, he gave to the scientific community a valuable EMG database, and interested researchers could measure their own EMG signals and compare them to the signals provided by Winter (on condition that they follow a similar experimental protocol as Winter). Since Winter is well-known scientist and one of the pioneers of human biomechanics research, we considered his EMG data to be properly recorded and valuable for comparison. Therefore, in order to test the efficacy of the system prototype proposed in this paper, we have also chosen the Winter EMG data base as an appropriate reference point to compare our measured data with. In order to obtain comparable data, we also followed the Winter guidelines for experiment setup in such a manner that we have used an electrode distance of 2 cm and have positioned electrodes as Winter explained (a description of the electrode positions is given in Table 1). We also calculated the envelopes from denoised and rectified signals, then we averaged envelopes over all measured subjects, and presented the averaged envelopes against the percentage of one gait cycle. Furthermore, we also divided gait cycle in 2% intervals, thus obtaining 51 samples for each EMG signal, like Winter. Therefore, our opinion is that comparison of our and Winter data is reasonable.

In order to provide quantitative measure about the difference between our measured EMG signals and signals provided by Winter, we calculated the RMSE, expressed by the Formula (19), where:

*x̂*[*n*]: samples of the average envelope of signals measured by our EMG system
*x*[*n*]: samples of the average envelope provided by Winter
*N*: number of envelope trajectory samples (*N* = 51)

## 4. EMG Signal Acquisition: Experiment Procedure

Ten volunteers (five male and five female), all students or employees at the University of Split, aged between 21 and 29 years (average = 24.6 ± 2.59 years), were recruited for the experiment. The purpose and procedures used in the experiment were thoroughly explained to the subjects and informed consent obtained. All subjects were healthy and did not suffer from any disease or malformation which might affect their motion patterns. Subjects were instructed to walk continuously on a horizontal laboratory surface at their most comfortable speed. EMG signals of six muscles from the most dominant activity during gait were recorded. The selected muscles were: gluteus maximus, biceps femoris (lateral hamstrings), sartorius, rectus femoris, tibialis anterior, and medial gastrocnemius. The electrode placements above the muscles were determined according to [29] (see Table 1). EMG activity during at least 10 gait cycles per subject was recorded.

## 5. Results and Discussion

### 5.1. Artificial EMG Signal Denoised by Wavelets

**Figure 11.**
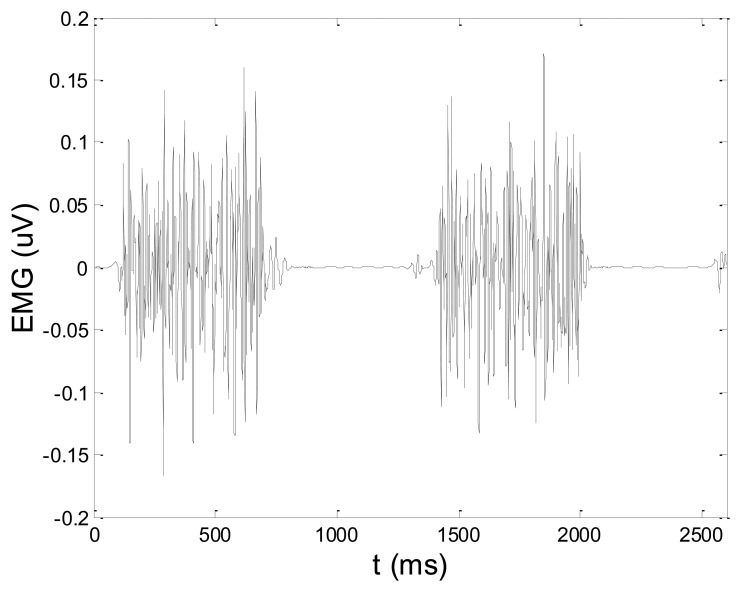
Simulated artificial EMG denoised by Daubechies5 wavelet and implementation of the soft sqtwolog threshold.

**Figure 12.**
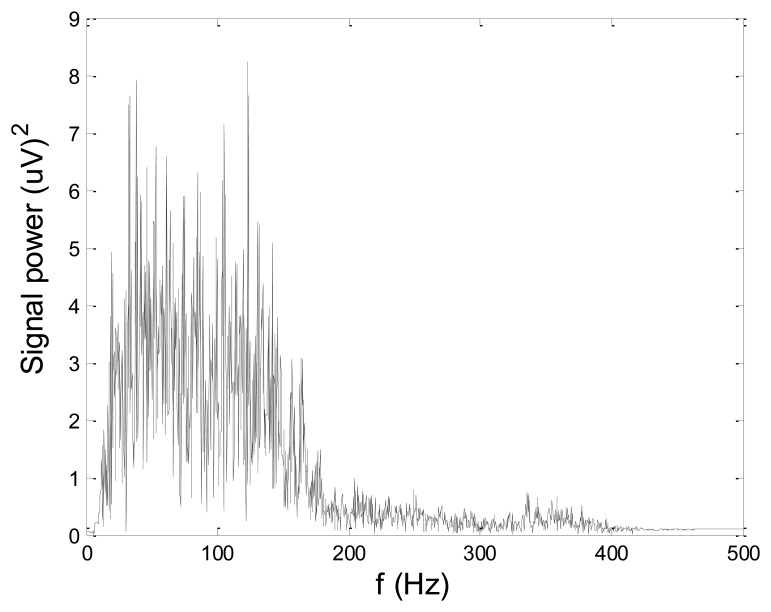
Power spectrum of the simulated artificial EMG denoised by Daubechies5 wavelet and implementation of the soft sqtwolog threshold.

Some of the results of wavelet-based denoising and denoising by classical filters are shown in Table 2. We show SNR and RMSE results for some of the tested wavelets, and the implementation of different thresholds. As can be observed from the table, wavelet-based denoising depends not only on the chosen wavelet, but also on the threshold as well. Classical filters (Butterworth and Chebyshev) provide much worse results in comparison with wavelets. The best results are obtained for Daubechies5 wavelet, and the implementation of the soft sqtwolog threshold. Also, by the visual inspection of denoised signals, we have concluded that the choice of Daubechies5 wavelet with the soft sqtwolog threshold gives the best results, among all. Figures 11 and 12 show a simulated EMG signal denoised by Daubechies5 wavelet with the soft sqtwolog threshold, and its power spectrum, respectively. From the Figures, the efficacy of the denoising procedure can be clearly observed. As can be seen, the noise from the periods with no EMG activity is almost completely removed. Therefore, we have chosen Daubechies5 wavelet, and the implementation of the soft sqtwolog threshold for denoising of real signals, measured by the prototype of the EMG system proposed in this paper.

Although we were doing off-line processing, we are aware of the fact that computation time of denoising algorithms is very important for some more complex applications such as online feedback and online control of prosthetic devices. Therefore, we measured computation time of Daubechies5 wavelet-based denoising of artificial EMG signal and compared it with computation times of classical filters. The obtained results are as follows: 14 ms for wavelet-based denoising and 16 ms for both Butterworth and Chebyshev filter.

### 5.2. Measured EMG Signals Denoised by Wavelets

**Figure 13.**
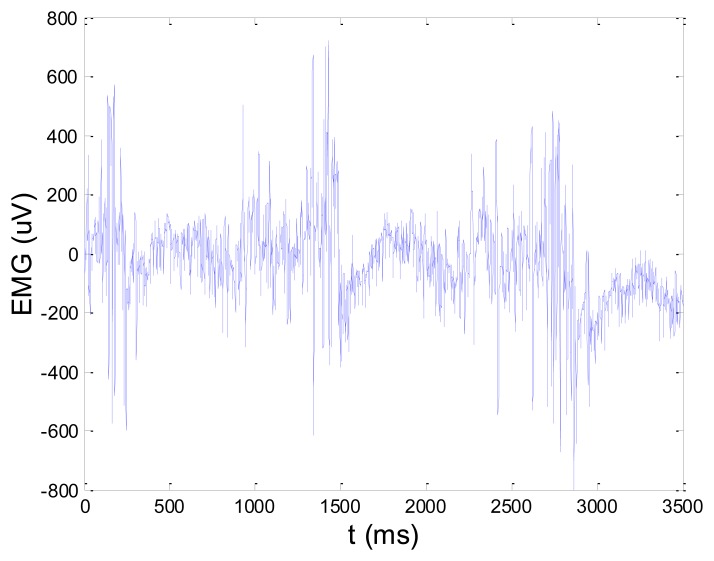
Raw EMG signal recorded on tibialis anterior muscle.

**Figure 14.**
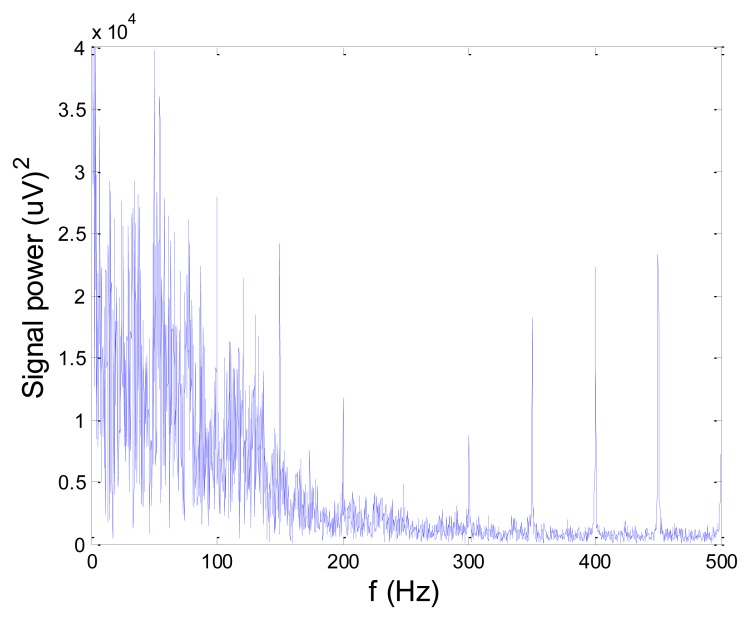
Frequency spectrum of raw EMG signal shown in Figure 13.

**Figure 15.**
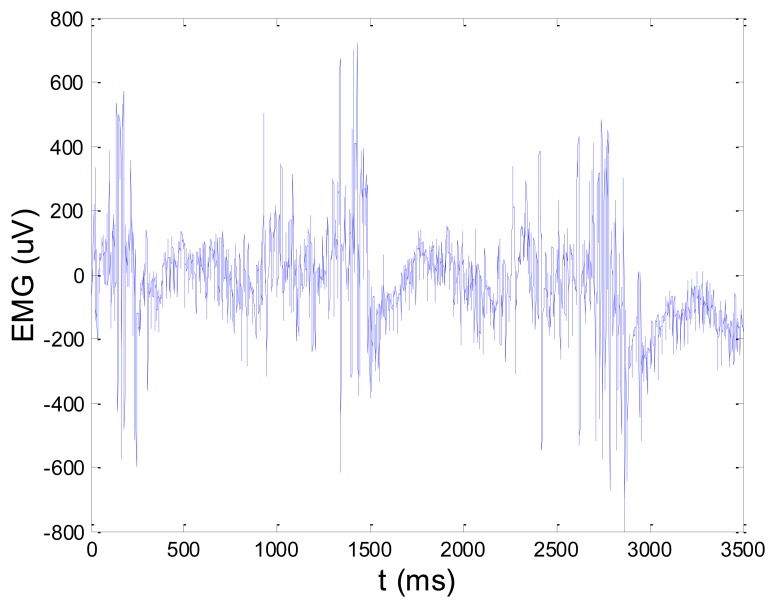
EMG signal (shown in Figure 13) filtered by wavelet method.

**Figure 16.**
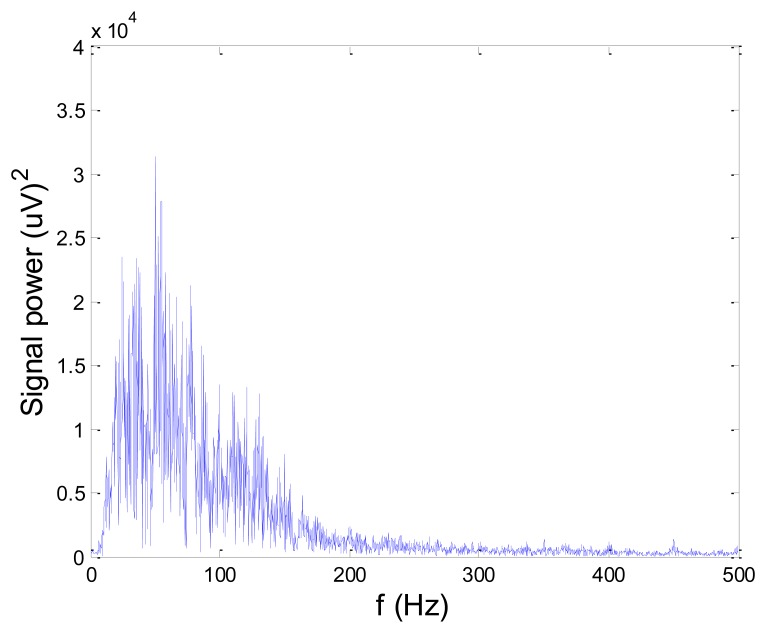
Frequency spectrum of signal filtered by wavelet method shown in Figure 15.

Figures 13 and 14 present an experimental raw myoelectric signal, recorded on the tibialis anterior muscle as described in Section 4, and its power spectrum, respectively. Our measurements showed that we have obtained the most noised signals measured on tibialis anterior. Therefore, we purposely showed signal measured on that muscle to present the efficacy of wavelet-based denoising. As can be seen from Figure 13, the signal is corrupted by motion artifact, white noise, and the accurate observation of muscle activity from the raw signal is barely possible. Also, if we look at the power spectrum in Figure 14, the presence of line interference on 50 Hz and higher harmonics is apparent. Figures 15 and 16 show the EMG signal (presented in Figure 13) denoised by wavelets and its power spectrum, respectively. From Figure 15 the efficacy of wavelet denoising can be noticed since the intervals of muscle activity and resting periods could be clearly observed.

Also, by observing the power spectrum of the denoised signal presented in Figure 16, the absence of noisy frequency components can be observed, even those of 50 Hz harmonics, since the good feature of wavelet–based denoising is that during the signal decomposition, the noisy coefficients are identified and eliminated prior the signal reconstruction. Therefore, we suggest wavelets as a powerful tool for EMG signal denoising.

### 5.3. EMG Activity of Dominant Muscles Recorded During Human Gait

**Figure 17.**
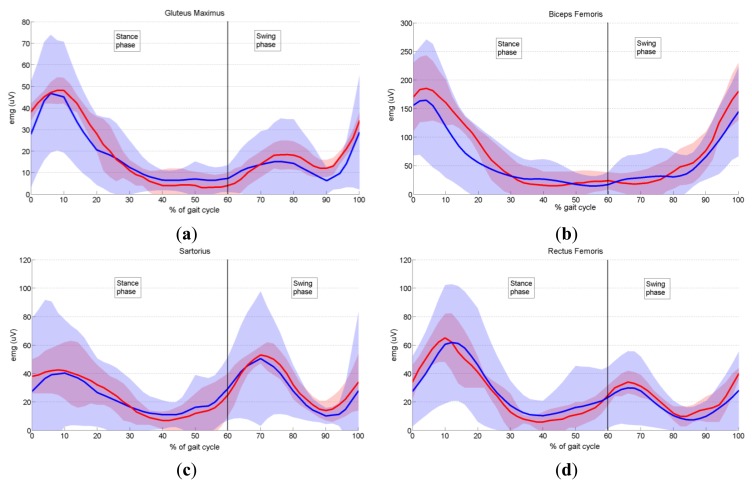

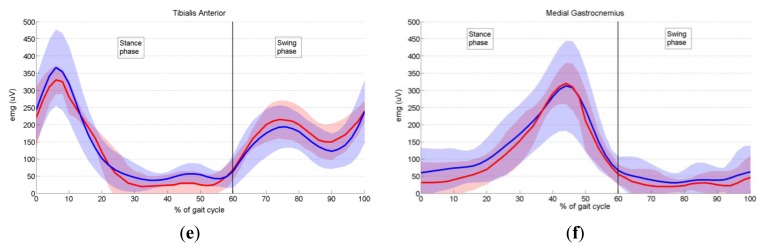
Average EMG activity of measured muscles during one gait cycle. Comparison of EMG data (± SD) measured by our EMG system (red lines ± red shaded area) and EMG reference data from the literature [29] (blue lines ± blue shaded area). (**a**) Average EMG activity of gluteus maximus during one gait cycle; (**b**) Average EMG activity of biceps femoris during one gait cycle; (**c**) Average EMG activity of sartorius during one gait cycle; (**d**) Average EMG activity of rectus femoris during one gait cycle; (**e**) Average EMG activity of tibialis anterior during one gait cycle; and (**f**) Average EMG activity of medial gastrocnemius during one gait cycle.

In order to validate the proposed prototype of EMG system, we measured the activity of six dominant muscles in ten healthy subjects during gait, and compared it with the EMG data provided by Winter [29], as explained in Section 3.6. Measured EMG signals from each muscle were denoised by wavelets, rectified and presented as envelopes. For each subject an EMG envelope of one complete gait cycle (0%–100% of gait cycle) was extracted. Envelope trajectories were averaged over all ten subjects and are represented in Figure 17 by thick red lines. Standard deviations (SD) of the envelopes are also presented (red shaded area). All data are plotted against the percentage of the completed gait cycle, thus making the gait cycle duration invariant. Thick blue lines (±SD, represented by the blue shaded area) in Figure 17 present EMG envelopes available in the literature [29]. By visual comparison of measured and referent envelope curves, we found fairly good overlap for all muscles. The discrepancy between our and referent data was further validated by calculating the quantitative measure, RMSE, as explained in Section 3.6. Table 3 presents obtained results. Furthermore, from Figure 17 it can be noticed that the standard deviations of our measured data have smaller values than those of the reference data. The possible explanation why we obtained smaller values of standard deviations could be the fact that Winter did his measurements on a larger number of subjects. Specifically, he measured the activity of gluteus maximus on 16 subjects, the activity of biceps femoris on 27 subjects, sartorius on 15, rectus femoris on 28, tibialis anterior on 26 and medial gastrocnemius on 25 subjects.

Let us briefly analyse the role of each measured muscle during gait cycle. In Figure 17a it can be seen that the major activity of gluteus maximus begins in late swing and peaks during weight acceptance (10% of stride), decreasing to a low level by the end of mid-stance (30% of gait cycle) [29]. A second minor burst of activity occurs during the first half of swing. The gluteus maximus is a hip extensor and acts during weight acceptance to control hip flexion.

The major activity of the biceps femoris (lateral harmstrings) (Figure 17b) begins in mid-swing (80% of stride) and continues into weight acceptance, peaking at 4% of stride. When heel contact occurs, the biceps femoris serves as a hip extensor to assist the gluteus maximus in controlling the forward rotation of the thigh.

Two equal peaks of activity are evident in the sartorius muscle (Figure 17c). The first peak occurs during weight acceptance (8% of gait cycle). It acts as a hip flexor, helping other hip extensors that are active at this time (biceps femoris, gluteus maximus). The second burst occurs early in swing (peaking at 70% of stride) and acts in a small way as a hip flexor to help swing the lower limb.

As regards the rectus femoris muscle (Figure 17d), one major and one minor burst of activity are present. The major activity begins before heel contact (90% of gait cycle) to extend the leg and foot just prior to heel contact and continues to a maximum during weight acceptance (10% of stride) when it acts as a knee extensor to control knee flexion. The second minor activity peaks just after toe-off (66% of stride), and has two simultaneous functions: hip flexion to pull the swinging limb forward, and knee extension to decelerate the backward swinging leg and foot.

Tibialis anterior, the ankle muscle (Figure 17e), commences its major activity at the end of swing to keep the foot dorsiflexed during the reach phase. Immediately after heel contact, it peaks (6% of gait cycle), which generate forces to lower the foot to the ground in opposition to the plantar flexing ground reaction forces. The second burst of activity commences at toe-off (60% of stride) and results in dorsiflexion of the foot for foot clearance during mid swing.

One major, long, phase of activity of the medial gastrocnemius muscle (Figure 17f) is evident, and it begins just prior to heel contact and rises during the stance phase, reaching a peak at mid push-off (44% of gait cycle). Until it reaches the peak, this muscle lengthens as the leg rotates forward under its control. The fine-tuning of this forward rotation is critical to knee flexion. After the peak has been reached, activity drops rapidly until toe-off (60% of stride), and low-level activity continues throughout the entire swing phase.

In conclusion, after comparing the close-matching measured and referent envelope trajectories, we can say that our EMG system performs well and can be used as an efficient tool for measurement of EMG activity during human motion.

## 6. Conclusions

In this paper we have described the design, development and testing of a prototype sEMG system. The intention was to provide detailed guidelines for researchers and engineers aiming to develop their own low-cost EMG systems applicable in biomechanical, clinical, rehabilitation, sport, and research contexts. We have suggested how to construct a bioamplifier suitable for recording low-amplitude biosignals such as EMG. Furthermore, we provided information on how to process EMG signals using wavelets in order to remove white noise, motion artifacts and line interference noise. We showed that wavelets, a state-of-the-art signal processing tool, provide better results in terms of signal denoising than classical digital filters.

Finally, we conducted an experiment in which we recorded EMG activity on ten healthy volunteers during gait. The aim was to record EMG activity in six dominant muscles during gait and to compare them with the EMG database available in literature [29]. The results show that our system is suitable for biomechanical and other applications.

Future plans include testing of the proposed system during more complex human motions such as sporting activities (cycling and rowing). Also, we plan to upgrade the existing prototype of the EMG system by developing a wireless system based on Bluetooth transmission [37], with higher sampling frequency (at least 2 kHz), and to implement more demanding EMG signal processing techniques like analysis of power spectrum with the intention to detect muscle fatigue and feature extraction for pattern recognition.

## Figures and Tables

**Table 1.** Surface electrode placement according to the Winter guidelines [29].

**Muscle**	**Surface Electrode Placement**
Gluteus maximus	Over the area of greatest muscle bulk proximal to a line between the greater trochanter and the ischial tuberosity
Biceps femoris (lateral hamstrings)	Midway on a line between the ischial tuberosity and the head of the tibula
Sartorius	Eight cm distal to the ASIS along a line to the medial epicondyle of the tibia
Rectus femoris	Midway between the ASIS and the superior border of the patella
Tibialis anterior	Over the area of greatest muscle bulk just lateral to the crest of the tibia on the proximal half of the leg
Medial gastrocnemius	Over the area of greatest muscle bulk on the medial calf

**Table 2.** Results of simulated EMG signal denoising.

**Wavelet/Filter**	**Threshold**	**Hard/Soft Thresh**	**SNR (dB)**	RMSE (*10^−3^)
Daubechies5	sqtwolog	soft	11.7591	8.90
Daubechies5	sqtwolog	hard	10.4864	10.20
Daubechies5	minimaxi	soft	9.6712	11.20
Daubechies5	minimaxi	hard	11.1132	9.57
Daubechies5	heursure	soft	11.3166	9.50
Daubechies5	heursure	hard	10.8425	9.91
Daubechies5	rigrsure	soft	11.3383	9.32
Symmlet8	sqtwolog	soft	7.8654	13.80
Symmlet8	sqtwolog	hard	10.8622	9.82
Symmlet8	minimaxi	soft	10.0175	10.81
Symmlet8	minimaxi	hard	11.0711	9.60
Symmlet8	heursure	soft	11.4907	9.10
Symmlet8	rigrsure	soft	11.4845	9.11
Coiflet5	sqtwolog	soft	8.2002	13.30
Coiflet5	sqtwolog	hard	10.9028	9.70
Coiflet5	minimaxi	soft	10.2323	10.50
Coiflet5	heursure	soft	11.6118	9.00
Coiflet5	rigrsure	soft	11.5581	9.03
Meyer	sqtwolog	soft	10.3258	10.35
Meyer	sqtwolog	hard	11.4132	9.15
Meyer	minimaxi	soft	11.3157	9.51
Meyer	minimaxi	hard	11.5743	9.01
Butterworth	–	–	3.5800	31.84
Chebyshew	–	–	2.6374	36.23

**Table 3.** RMSE errors between envelopes obtained from our measured EMG signals and envelopes provided by Winter [29].

**Muscle**	**RMSE (**μ**V)**
Gluteus maximus	4.3218
Biceps femoris	21.1814
Sartorius	4.3119
Rectus femoris	5.1810
Tibialis anterior	21.8500
Medial gastrocnemius	20.1229
