# Correction: Supuk, T.G., *et al.* Design, Development and Testing of a Low-Cost sEMG System and Its Use in Recording Muscle Activity in Human Gait. *Sensors* 2014, *14*, 8235–8258

**DOI:** 10.3390/s140815639

**Published:** 2014-08-22

**Authors:** Tamara Grujic Supuk, Ana Kuzmanic Skelin, Maja Cic

**Affiliations:** Faculty of Electrical Engineering, Mechanical Engineering and Naval Architecture, Laboratory of Biomechanics and Automatic Control Systems, R. Boskovica 32, Split 21000, Croatia; E-Mails: akuzmani@fesb.hr (A.K.S.); maja@fesb.hr (M.C.)

The authors wish to make the following correction to this paper [1]. Due to an error Figure 15 was a duplicate of Figure 13, the former Figure 15 (labelled here as Previous Figure 15) should be replaced by the new version shown below (labeled here as New Figure 15):

We apologize for any inconvenience caused to the readers.

## Figures and Tables

**Previous Figure 15.**
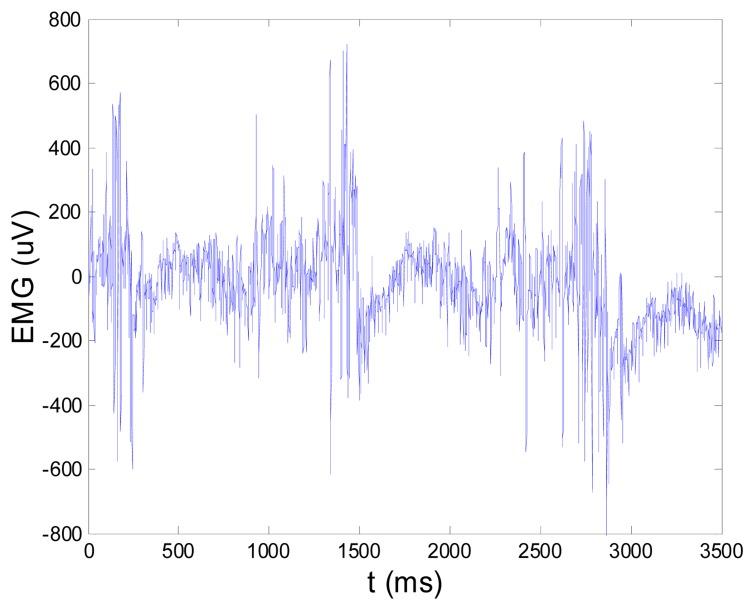
EMG signal (shown in Figure 13) filtered by wavelet method.

**New Figure 15.**
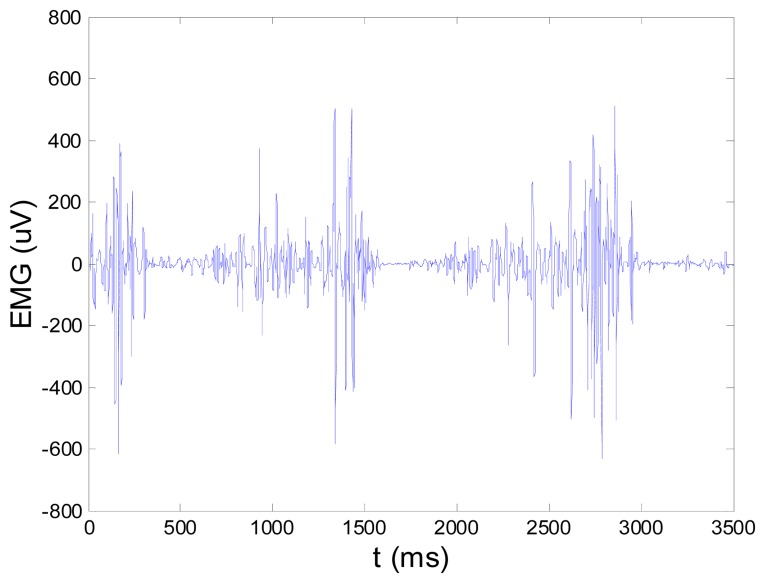
EMG signal (shown in Figure 13) filtered by wavelet method.
